# Factors affecting preventive behaviors of Alzheimer’s disease in family members of patients with Alzheimer’s disease

**DOI:** 10.1097/MD.0000000000031136

**Published:** 2022-10-21

**Authors:** JiEun Kim, Min Young Kim, Jung-Ae Kim, Youngeun Lee

**Affiliations:** a Medical, Eisai Korea Inc, Gangnam-gu, Seoul, Republic of Korea; b Real World Solutions, IQVIA Korea, Jung-gu, Seoul, Republic of Korea.

**Keywords:** Alzheimer disease, dementia, dementia preventive behavior, family members of AD patients, health belief model

## Abstract

As genetic factors increase the risk of Alzheimer’s disease (AD), the families of dementia patients are at risk of AD. We aimed to evaluate the factors affecting preventive behaviors of AD in family members of AD patients. Using constructed questionnaire based on the Health Belief Model (HBM) theoretical framework, this cross-sectional study investigated factors influencing preventive behaviors of AD such the intention to take AD-preventive medicines, prior experience of taking cognitive function supplements, and AD-preventive lifestyle. 147 family members of AD patients were recruited through the Korea Alzheimer’s Caregiver Association. Out of 147 participants, 94.6% had intention to take AD-preventive medicines and 46.3% had experience of taking cognitive function supplements. The intention to take AD-preventive medicines were significantly influenced by self-efficacy (odds ratio [OR] 1.39, 95% confidence interval [CI] 1.03, 1.87) and dementia knowledge (OR 3.42, 95% CI 1.13, 10.39), whereas prior experience of cognitive function supplements was significantly associated with cue to action (OR 1.22, 95% CI 1.07, 1.40). AD-preventive lifestyle was significantly influenced by socio-demographics such as age, sex, and marital status, and the HBM factors such as perceived susceptibility, self-efficacy, and cue to action. Self-efficacy, cue to action, dementia knowledge, and perceived susceptibility were significantly associated with preventive behaviors of AD. Also, family members of dementia patients, who are at risk of dementia due to genetic factors, lifestyles, and environment factors, had high level of AD-preventive lifestyle and strong intention to take AD-preventive medicines. Further research could be suggested to understand AD-preventive behavior and intention to take AD-preventive medicines in general population.

## 1. Introduction

With the rapid growth of an aging population, the worldwide prevalence of dementia has also increased rapidly. It is estimated that approximately 50 million patients are diagnosed with dementia worldwide and about 10 million new cases have been reported annually.^[1]^ In South Korea, the estimated prevalence of dementia is also expected to continuously increase until 2050, reaching 15% to 16%.^[2,3]^

The families of patients with dementia are at risk of dementia due to genetic factors, lifestyle, and environmental factors.^[4–6]^ Previous studies have reported that dietary, lifestyle, and medical conditions were associated with the risk of Alzheimer’s disease (AD) and cognitive decline. Wang et al also reported that early-life factors such as food deficiency, education level, and family-related factors were associated with the risk of dementia and cognitive impairment in later life. In addition, the genetic factors, particularly the apolipoprotein E gene variation, were reported to be associated with increased risk of AD. Further, family members of patients with dementia are also expected to have higher level of dementia knowledge and stronger perceptions and personal beliefs regarding dementia in terms of susceptibility, severity, benefits, and barriers compared to the general population.

Therefore, identifying factors that significantly influence on health behaviors to prevent AD among those at risk of dementia would be important in establishing dementia prevention policies for the general public. However, most previous studies have been conducted on the elderly population, and none of the studies evaluated health behaviors associated with dementia prevention in family members of patients with dementia in South Korea. This study aimed to assess dementia preventive health behaviors of family members who care for patients with dementia, and to identify factors influencing on their health behaviors.

## 2. Methods

### 2.1. Theoretical framework

Health behaviors associated with dementia prevention include screening for dementia, taking brain function enhancing supplements, regular exercise, smoking cessation, and abstaining from alcohol, and the practice of such health behaviors can be influenced by variety of factors. Therefore, this study adopted the health belief model (HBM) as theoretical framework and used questionnaires based on the HBM to identify the factors affecting the preventive health behaviors of AD.

The HBM is a model that predicts and explains health behaviors related to disease prevention and the influencing factors, and has been widely applied to the analysis of factors influencing the practice of preventive health behaviors.^[7,8]^ In addition, Galvin et al^[9]^ developed a questionnaire that can evaluate knowledge of dementia and factors influencing behavior for dementia screening based on this HBM. According to previous studies based on the HBM, demographic factors including sex, age, marital status, screening experience of close acquaintances, level of education, and smoking status had a significant effect on preventive behaviors of AD such as screening tests. For the factors related to the HBM, higher perceived susceptibility, perceived severity, perceived benefits, self-efficacy, and lower perceived barriers promoted health behaviors that are associated with dementia prevention, however, inconsistency in statistical significance was observed across studies.^[10–13]^ Therefore, this study investigated both socio-demographics and health beliefs as key mediators of intention to engage in behaviors to reduce the risk of dementia development.

### 2.2. Study design

A cross-sectional study was conducted to evaluate preventive behaviors of AD and factors affecting those health behaviors among family members of patients with AD. Face-to-face interviews with a constructed questionnaire based on the HBM theoretical framework were conducted.

#### 2.2.1. Participants and recruitment.

Family members of patients with AD were recruited from January 20, 2020 to March 31, 2020 through the Korea Alzheimer’s Caregiver Association. Eligible criteria for this study were: family members of the patients who were diagnosed with dementia due to AD; aged between 45 and 65 years (as of the year of the survey); residents of Seoul, other metropolitan cities (Gyeonggi-do and Incheon), and other areas (near Daejeon, Daegu, Busan, and Kwangju); be able to read or write Korean; and be able to provide informed consent. In total, 147 subjects were eligible to participate, and all study participants provided informed consent. This study was approved by the Korea National Institute for Bioethics Policy designated by the Korea Ministry of Health and Welfare (P01-201912-21-004). The sample size for this study was calculated as 128 subjects for conducting a 2-sided test with power of 80%, alpha level of 0.05, and effect size of 0.5, and the survey was conducted on 147 subjects considering 15% potential incompletion rate in some questions.

#### 2.2.2. Procedure.

Each interview was conducted in a meeting room with one or two trained research staff members and lasted for approximately 60 minutes. Prior to commencing an interview, research staffs provided explanation on the purpose of this study, the approximate time required to complete the questionnaire, and obtained written informed consent from all study participants. The interviews were audio-recorded with consent from all study participants. Participants were then asked to complete the questionnaire for this study. Additionally, we collected information on the characteristics of study participants including socio-demographics, relationship with patients with AD, and health literacy. The health literacy was assessed by using a shortened form of the Korean Health Literacy Scale, consisting of 7 comprehension and numeracy questions and 5 health-related questions.^[14]^ Further, the pretest interview was performed to assess the validity of survey questionnaires and to identify potential limitations or obstacles with regards to research methodology, management of the entire survey process, and word ambiguity.

### 2.3. Measures

#### 2.3.1. Socio-demographic characteristics.

The following items were collected to understand the general socio-demographic characteristics of the participants of this study: sex, age, residence, marital status, level of education, and level of income, and relationship with AD patients. The income level was converted from South Korean Won to US Dollar with the average foreign exchanges rate in the year of 2020 (1 US Dollar = 1250 Korean Won).^[15]^

#### 2.3.2. Preventive behavior of AD.

We evaluated preventive behaviors of AD in terms of drug-related behaviors and lifestyle-related behaviors. The drug-related behaviors were evaluated based on the intention to take AD-preventive medicines and prior experience of taking cognitive function supplements. The intention and prior experience were assessed by asking participants’ willingness to take medication that prevents AD and worsening of symptoms if developed in the future or participants’ experience of taking cognitive function enhancing supplements within the last 3 years. The questionnaires were made up of binary questions with the 2 possible answers, “Yes” or “No.” The lifestyle-related behaviors were evaluated using 9 items to measure participants’ daily lifestyle that can prevent AD. The items included having regular meals, having well-balanced nutrition, cessation of alcohol drinking and smoking, managing body weight, having regular exercise, having regular intellectual activities (e.g., reading, watching movie, etc), managing chronic disease regularly, and frequently interacting with family members or friends. Each item was rated on a 5-point Likert scale ranging from “strongly disagree” to “strongly agree.”

#### 2.3.3. Factors affecting health behaviors to reduce the risk of dementia.

Based on the HBM as conceptual framework, perceived susceptibility (if they think they are vulnerable to developing AD), perceived severity (if they identify the seriousness or severity of developing AD), perceived benefits (if they believe the health behaviors available to be effective in reducing the risk of AD), perceived barriers (if they identify the negative attributes or obstacles related to the health behaviors), self-efficacy (confidence in their abilities to successfully complete the health behaviors), cue to action (factors triggering engagement in the health behaviors), and knowledge of dementia were assessed as potential factors affecting health behaviors that reduce the risk of dementia.

The domain for perceived susceptibility (4 items), perceived severity (4 items), perceived benefits (4 items), perceived barriers (11 items), and self-efficacy (7 items) were measured using the Intention to Screen Questionnaire, developed by Galvin et al^[9]^ to understand the psychosocial factors of the dementia screening test. An approval for the use of questionnaire was obtained from the original author. Each item was rated on a 5-point Likert scale ranging from “strongly disagree” to “strongly agree.” Cue to action was measured using 5 items with a 5-point Likert scale to assess specific cues that can have an impact on the actions taken for AD prevention. The knowledge of dementia was measured using 12 items with response options, “correct” or “incorrect.” Each domain score was calculated by taking the sum of items. A higher score indicated participant’s belief that they were at high risk, expected benefits and few barriers to action, had self-confidence to action, had adequate knowledge about AD, and AD was severe disease. The survey tool demonstrated adequate reliability with Cronbach’s α of 0.60 or higher: 0.887 for perceived susceptibility, 0.697 for perceived severity, 0.772 for perceived benefits, 0.785 for perceived barriers, 0.867 for self-efficacy, and 0.707 for cue to action.^[16]^

### 2.4. Statistics

Descriptive analysis was used to assess participant characteristics and scores from the survey. Descriptive statistics of intergroup comparison was done using t-test for continuous variables and chi-square test for categorical variables. A multiple logistic regression was used to identify factors affecting the intention to take AD-preventive medicines and experience of taking cognitive function enhancement supplements, and a generalized linear regression for factors affecting the AD-preventive lifestyle. For the multivariate regression analysis, socio-demographic characteristics (sex, age, residence, level of education, marital status, and household income), HBM variables, and overall dementia knowledge scores were included as independent variables. All statistical tests were 2-sided with a significance level of 0.05. The statistical analysis was performed using SAS 9.3 (SAS Institute, Cary, NC).

## 3. Results

### 3.1. Participants characteristics

One hundred and forty-seven subjects were recruited and completed the survey, with an average age of 55.2 ± 5.2 years. A significant proportion of participants were female (68%), married (93.9%), and had a college degree or a higher (70.8%). No significant differences were observed in socio-demographic characteristics according to the intention to take AD-preventive medicines and prior experience of taking cognitive function supplements. In contrast, AD-preventive lifestyle was significantly different according to sex and age. Females (33.7 ± 5.1) showed significantly higher scores of AD-preventive lifestyle compared to males (32.0 ± 4.2) (*P* = .05). The 54 to 59 years age group (31.6 ± 4.9) showed the lowest score among the age groups divided in 5-year intervals (Table 1).

**Table 1:** Socio-demographics of participants.

	Total	Intention to take AD-preventive medicines			Experience of taking Gingko biloba or similar medicines			AD-preventive lifestyle score	
		Yes	No	*P* value	Yes	No	*P* value	Mean(±SD)	*P* value
Total	147	139 (94.6)	8 (5.4)		68 (46.3)	79 (53.7)		33.2 (±4.9)	
Sex									
Male	47 (32.0)	43 (30.9)	4 (50.0)	.261‡	26 (38.2)	21 (26.6)	.131‡	32.0 (±4.2)	.050‡
Female	100 (68.0)	96 (69.1)	4 (50.0)		42 (61.8)	58 (73.4)		33.7 (±5.1)	
Age									
Mean (±SD)	55.2 (±5.2)	55.3 (±5.2)	53.5(±4.0)	.337†	55.2 (±5.3)	55.2 (±5.1)	.966†	55.2 (±5.2)	-
45-49	24 (16.3)	23 (16.6)	1 (12.5)	.547‡	11 (16.2)	13 (16.5)	.116‡	34.5 (±5.0)	.050‡
50-54	41 (27.9)	37 (26.6)	4 (50.0)		24 (35.3)	17 (21.5)		33.8 (±4.2)	
54-59	48 (32.7)	46 (33.1)	2 (25.0)		16 (23.5)	32 (40.5)		31.6 (±4.9)	
60-64	34 (23.1)	33 (23.7)	1 (12.5)		17 (25.0)	17 (21.5)		33.6 (±5.1)	
Residence									
Metropolitan	89 (60.5)	85 (61.2)	4 (50.0)	.418‡	45 (66.2)	44 (55.7)	.195‡	33.1 (±5.0)	.821‡
Others	58 (39.5)	54 (38.9)	4 (50.0)		23 (33.8)	35 (44.3)		33.3 (±4.6)	
Education									
≤High school	43 (29.3)	40 (28.8)	3 (37.5)	.796‡	18 (26.5)	25 (31.6)	.492‡	32.3 (±4.7)	.156‡
≥University	104 (70.8)	99 (71.2)	5 (62.5)		50 (73.5)	54 (68.4)		33.5 (±4.9)	
Marriage									
Yes	138 (93.9)	131 (94.2)	7 (87.5)	.439‡	63 (92.6)	75 (94.9)	.564‡	33.3 (±4.9)	.191‡
No	9 (6.1)	8 (5.8)	1 (12.5)		5 (7.4)	4 (5.1)		31.1 (±4.5)	
Monthly income*									
<3200 USD	35 (23.8)	32 (23.0)	3 (37.5)	.350‡	12 (17.6)	23 (29.1)	.104‡	32.1 (±4.4)	.133‡
≥3200 USD	112 (76.2)	107 (77.0)	5 (62.5)		56 (82.4)	56 (70.9)		33.5 (±5.0)	

Values are presented as n (%).

AD = Alzheimer’s disease, SD = standard deviation, USD = US Dollar.

* Income level was converted from Korean Won (KRW) to USD with the 2020 average currency rate of 0.0008 USD. The median household income for 4-person families were 3799.3 USD in 2020.^[26]^

† *t* test.

‡ Chi-square test.

### 3.2. Preventive behaviors of AD

**Figure 1.**
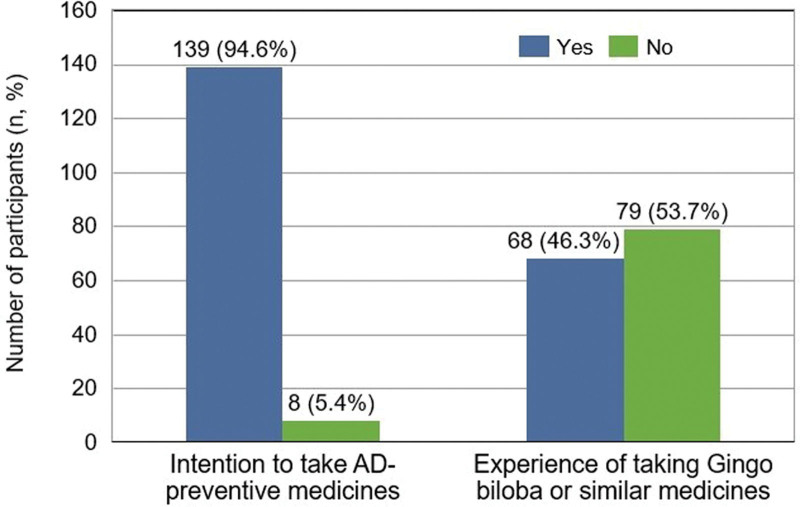
Response on AD-preventive medicines. Values are presented as n (%). AD = Alzheimer’s disease.

With regard to AD-preventive medicines, 137 (94.6%) had the intention to take AD-preventive medicines and 68 (46.3%) had the prior experience of taking cognitive function supplements among 147 participants (Fig. 1).

The average total score of AD-preventive lifestyle was 33.2 out of 45 points and the average item score was 3.7 out of 5 points. Among the lifestyle items, the highest score was reported in smoking cessation (4.4) and the lowest score was observed in regular intellectual activities such as reading and watching movies (3.2). The total score of dementia-risk reducing lifestyle habits was higher in the participants with the intention to take AD-preventive medicines (33.3 vs 30.5) and those with prior experience of taking cognitive function supplements (33.8 vs 32.6) compared to those who did not have an intention or prior experience; however, no significant differences were observed between 2 groups. Of 9 items related to lifestyle behaviors, participants with the intention (3.7 vs 2.8) or prior experience (3.8 vs 3.5) scored significantly higher in regular management of chronic disease compared to those without the intention or experience. Although no significant differences were observed in the other items, the participants who had the intention or prior experience tended to report higher scores than those who did not (see Table, Supplemental Content 1, http://links.lww.com/MD/H613).

### 3.3. Health beliefs and dementia knowledge

The total score of HBM (107.2 vs 97.1) and dementia knowledge (108.7 vs 104.8) were significantly higher in participants who had the intention to take AD-preventive medicines or prior experience of taking cognitive function supplements compared to those who did not. Among the domains of HBM, self-efficacy showed significantly higher score in both participants who had the intention or prior experience, whereas perceived susceptibility and cue to action were significantly higher in participants with prior experience compared to those without the experience. The knowledge of dementia was significantly higher in participants with the intention, whereas no significant differences were observed between patients who did or did not have the prior experience (Table 2).

**Table 2:** Score of Alzheimer’s disease (AD) preventive behaviors related to drugs according to the intention and prior experience.

	Score range	Total (n = 147)	AD-preventive behaviors related to medicines					
			Intention to take AD-preventive medicines			Experience of taking Gingko biloba or similar medicines		
			Yes (n = 139)	No (n = 8)	*P* value	Yes (n = 68)	No (n = 79)	*P* value
**HBM variables**								
Total score	30-150	106.6 (11.0)	107.2 (10.7)	97.1 (12.0)	.012†	108.7 (10.8)	104.8 (10.8)	.029†
Perceived susceptibility	4-20	12.9 (3.6)	13.0 (3.5)	10.6 (3.7)	.068†	13.7 (3.5)	12.2 (3.5)	.010†
Perceived severity	4-20	15.7 (2.8)	15.7 (2.7)	14.3 (3.3)	.142†	16.0 (2.8)	15.3 (2.8)	.143†
Perceived benefits	4-20	16.5 (2.4)	16.5 (2.4)	15.5 (2.5)	.247†	16.8 (2.4)	16.2 (2.5)	.172†
Perceived barriers	11-55	34.9 (6.4)	34.9(6.5)	34.6 (4.2)	.914†	34.6 (6.6)	35.1 (6.3)	.630†
Self-efficacy	7-35	26.8 (4.5)	27.0 (4.4)	22.1 (4.8)	.003†	27.7 (4.4)	26.0 (4.5)	.020†
Cue to action	5-25	12.7 (3.4)	12.8 (3.5)	10.8 (2.0)	.096†	13.8 (3.7)	11.8 (3.0)	.000†
**Non-HBM variables**								
Knowledge of dementia	0-12	9.7 (1.3)	9.7 (1.2)	8.13 (1.4)	.001†	9.7 (1.2)	9.6 (1.3)	.518†

Values are presented as mean (SD).

† *t* test

AD = Alzheimer’s disease, HBM = health belief model, MCI = mild cognitive impairment, SD = standard deviation.

### 3.4. Analysis of factors affecting preventive behaviors of AD

In multiple regression analysis, the factors significantly affecting the intention to take AD-preventive medicines were self-efficacy (odds ratio [OR] 1.39, 95% confidence interval [CI] 1.03, 1.87) and knowledge of dementia (OR 3.42, 95% CI 1.13, 10.39), whereas cue to action (OR 1.22, 95% CI 1.07, 1.40) was significantly related to the prior experience of taking cognitive function supplements. The generalized linear regression analysis identified that the dementia risk-reducing lifestyle habits were significantly influenced by participants’ socio-demographics such as age, sex, and marital status, and the HBM factors such as perceived susceptibility, self-efficacy, and cue to action. Female (β = 1.73, 95% CI 0.14, 3.32) had positive impact on the dementia risk-reducing lifestyle habits, whereas age in 50-55 (β = -3.06, 95% CI -5.38, -0.75) and unmarried status (β = -3.96, 95% CI -5.38, -7.15, -0.76) negatively affected the risk-reducing lifestyle habits. Among the domain of HBM, perceived susceptibility (β = -0.35, 95% CI -0.59, -0.11) showed negative impact on the risk-reducing lifestyle habits, whereas self-efficacy (β = 0.18, 95% CI 0.00, 0.36) and cue to action (β = 0.25, 95% CI 0.02, 0.48) had positive impact on the risk-reducing lifestyle habits (Table 3).

**Table 3:** Analysis of factors affecting Alzheimer’s disease (AD) preventive behaviors.

	Reference	Intention to take AD-preventive medicines	Experience of taking Gingko biloba or similar medicines	AD-preventive lifestyle
		OR (95% CI)	OR (95% CI)	B (95% CI)
**Socio-demographic factors**				
Female	Male	9.08 (0.65-127.78)	0.35 (0.14-0.83)	1.73 (0.14-3.32)
Age in 50-54	45-49	0.16 (0.01-4.83)	1.64 (0.52-5.13)	-0.81 (-3.00-1.38)
Age in 55-59	45-49	0.41 (0.01-24.12)	0.51 (0.15-1.71)	-3.06 (-5.38 - -0.75)
Age in 60-64	45-49	0.78 (0.01-46.85)	0.90 (0.26-3.14)	-1.13 (-3.51-1.24)
Residence: Others	Metropolitan	2.14 (0.20-23.16)	0.75 (0.33-1.71)	0.85 (-0.72-2.42)
Education: ≥university	<high school	6.65 (0.36-121.82)	0.61 (0.24-1.59)	1.05 (-0.76-2.87)
Unmarried	Married	0.02 (0.00-2.02)	1.23 (0.22-6.73)	-3.96 (-7.15 - -0.76)
Monthly income*: ≥3200 USD	< 3200 USD	1.58 (0.15-16.88)	2.07 (0.77-5.55)	0.70 (-1.12-2.51)
**Non-demographic factors**				
Perceived susceptibility	+1 score	1.20 (0.83-1.72)	1.03 (0.90-1.17)	-0.35 (-0.59 - -0.11)
Perceived severity	+1 score	1.02 (0.70-1.49)	1.00 (0.86-1.17)	-0.16 (-0.45-0.13)
Perceived benefits	+1 score	1.06 (0.61-1.84)	1.07 (0.89-1.27)	-0.02 (-0.34-0.31)
Perceived barriers	+1 score	1.07 (0.82-1.39)	1.01 (0.94-1.08)	0.08 (-0.05-0.21)
Self-efficacy	+1 score	1.39 (1.03-1.87)	1.07 (0.98-1.18)	0.18 (0.00-0.36)
Cue to action	+1 score	1.21 (0.73-2.02)	1.22 (1.07-1.40)	0.25 (0.02-0.48)
Knowledge of dementia	+1 score	3.42 (1.13-10.39)	1.06 (0.78-1.44)	0.13 (-0.44-0.70)

* Income level was converted from Korean Won (KRW) to USD with the 2020 average currency rate of 0.0008 USD. The median household income for 4-person families were 3799.3 USD in 2020.^[26]^

AD = Alzheimer’s disease, CI = confidence interval, OR = odds ratio, USD = US Dollar.

OR was derived from a multiple regression analysis; B was derived from a generalized linear regression.

## 4. Discussions

This study aimed to evaluate the practice of preventive behaviors of AD in family members of patients with AD, and to analyze the factors affecting its practice. We found that the practice level of preventive behavior of the participants was high; self-efficacy, cue to action, and dementia knowledge mainly affected their intention to take AD-preventive medicines in the future and the prior experience of taking cognitive function supplements; and the sex, age, marital status, perceived susceptibility, self-efficacy, and cue to action affected AD-preventive lifestyle.

Most previous studies that assessed the preventive behavior of AD in South Korea were conducted for the elderly population.^[10,12,17]^ To our knowledge, this is the first survey-based study that assessed the factors affecting preventive behaviors of AD among family members of AD patients, with an average age of 55 years, in South Korea. As the family history and increasing age are known risk factors of developing dementia.^[5,18]^ and middle-aged people in their mid-50s are the age group that prepares to enter elderhood, the participants for this study might be considered adequately selected group for assessing preventive behaviors of AD.

In the present study, age, sex, marital status, perceived susceptibility, self-efficacy, and cue to action significantly affected dementia risk-reducing lifestyle habits. Approximately 95% of the study participants answered “Yes” to the item asking about their intention to take AD-preventive medicines in the future when it is developed, indicating high unmet needs for AD-preventive medicines. However, only 46.3% of the participants had an experience of taking cognitive function enhancement supplements, in contrast to the high proportion of the positive answers for the intention to take AD-preventive medicines. It can be presumed that these differences were caused by different levels of expectation for the AD preventive effect of both medicines. The survey was conducted based on the assumption that AD-preventive medicines are the substances with actual preventive effect, whereas cognitive function enhancement supplements such as ginkgo biloba extract can be considered as functional food. According to a previous study that examined the effect of ginkgo biloba extract, the incidence rate of dementia was 3.3 and 2.9 per 100 population in the ginkgo biloba extract group and placebo group, respectively.^[19]^ No significant difference in the incidence rates of dementia was observed between 2 groups. In addition, Snitz et al^[20]^ reported that the rate of decline in cognitive function was not significantly decelerate, thus the effect of ginkgo biloba on the improvements in cognitive function was not observed. If AD-preventive medicines with proven efficacy became available in the future, the willingness to take them is expected to be very high among those at risk of dementia.

In addition, the level of practicing AD-preventive lifestyle was also higher in this study compared to the findings from previous studies that assessed general elderly population. Kim et al^[17]^ reported that the average AD-preventive activity score was 64 points out of 100 points for the elderly in urban areas and 63 points for the elderly in rural areas. In the present study, the average score was 74 points out of 100 points. Considering the differences in survey questionnaires between Kim et al and the present study, the 10-point difference between 2 studies is considered to be significant. This indicates that the participants included in this study may have higher interest in dementia prevention compared to other elderly population. Among those with high interest, the most important factors affecting the preventive behaviors of AD were identified as self-efficacy and cue to action. Significant association was observed between these 2 factors and measurements related to preventive behaviors of AD.

In the factor analysis, higher self-efficacy was significantly more likely to receive AD-preventive medicines compared to those who had low self-efficacy. Prior experience in taking cognitive function supplements was also associated with use of AD-preventive medicines with by 1.07-fold, however, no statistical significance was observed. These findings were consistent with the previous studies. Yoo et al^[10]^ found that for every 1-point increase in the self-efficacy score, the probability of taking dementia screening test significantly increased by 2.85 (OR 2.85, 95% CI 1.46 5.57). Further, Choi et al^[12]^ reported that self-efficacy was the most significant factor influencing the intention to prevent dementia (β = 0.38, *P* < .001). This supports the assumption of the HBM and social cognitive theory that the higher the confidence in the health behavior performance ability, the better the preventive health behaviors are performed.^[7,21]^ Therefore, these factors need to be considered for planning dementia prevention policies in the future. Cue to action showed positive association with intention to take AD-preventive medicines, prior experience of taking medicines, and AD-preventive lifestyle, however, statistical significance was observed in the latter 2 behaviors. The impact of cue to action varied across studies. According to Werner, cue to action was found to be related to the health behaviors for preventing dementia.^[13]^ However, Choi et al^[12]^ reported no significant relationship between cue to action and intention of AD-preventive behaviors. We found that perceived susceptibility, age, and marital status affected the dementia preventive lifestyle habits whereas the intention of AD-preventive behaviors was influenced by the knowledge of dementia. These findings could be interpreted that the influencing factors could be differed depending on the evaluation items of dementia prevention health behavior. Moreover, our results on the effects of the HBM factors on preventive behaviors of AD were found to be inconsistent with those observed in previous studies. These conflicting results could potentially be due to the cultural differences and the survey effect. Factors influencing beliefs and motivations may differ between cultures.^[22]^ Differences in study subjects, research methods and interviewers could have an impact on the survey response, leading to inconsistencies in results across studies.^[23]^

Findings of this study suggest following aspects to be considered when developing government policies and interventions for dementia prevention. First, the most important factors influencing preventive behaviors of AD and beliefs for each prevention program should be identified separately. Second, self-efficacy enhancement interventions or programs should be prioritized. Third, educational programs should be organized to improve cue to action and dementia awareness. Last, the high-risk group for dementia should be continuously managed in appropriate and effective ways. The elderly and the family members of patients with dementia are representative high-risk groups, and in this study, the family members of dementia patients were highly motivated to practice activities to prevent dementia. However, previous studies reported that most of the high-risk groups did not appear to have dementia preventing behaviors due to an increased burden for caregivers.^[24]^ Therefore, policies and interventions should prioritize family members of patients with dementia.

There are several limitations to be considered when interpreting our results. The participants of this study are family members of patients with dementia, which may not represent the general population of the same age group in South Korea. The family members of patients with dementia may have different perceptions of dementia from the general population. It has been reported that family members of dementia patients have significantly higher perceived susceptibility (12.2 vs 10.2) and perceived severity (15.6 vs 13.7) compared to the non-family group.^[25]^ Therefore, generalization of the findings should be done with consideration of these limitations. Furthermore, the intention to take AD-preventive medicines, which was not available at the point of survey, was intended to evaluate participants’ willingness to consume AD-preventive medicines in the future based on the information given during the survey. As a result, their responses could change after the actual release of the AD-preventive medicines depending on the given information such as safety, efficacy, and insurance coverage. However, it may be meaningful in that it has provided a criterion for evaluating the unmet medical need for AD-preventive medicines of the participants.

In the present study, the participants showed a high level of dementia risk-reducing health lifestyle habits and had strong willingness to take AD-preventive medicines in the future. Self-efficacy, cue to action, knowledge of dementia, and perceived susceptibility were significantly associated with preventive behaviors of AD. Consequently, it is necessary to implement policies that can improve the level of health belief and education on dementia. Findings of this study could be used as an evidence in developing policies and interventions preventing dementia.

## Author contributions

JiEun Kim, Jung-Ae Kim, Min Young Kim, and Youngeun Lee designed the study. Jung-Ae Kim and Youngeun Lee performed face-to-face interview. JiEun Kim, Jung-Ae Kim and Youngeun Lee analyzed the data. JiEun Kim, Jung-Ae Kim, and Min Young Kim interpreted the data and wrote the manuscript. All authors have reviewed and approved the final version of the manuscript.

**Conceptualization:** JiEun Kim, Min Young Kim, Jung-Ae Kim.

**Data curation:** Jung-Ae Kim, Youngeun Lee.

**Formal analysis:** JiEun Kim, Jung-Ae Kim.

**Methodology:** JiEun Kim, Min Young Kim, Jung-Ae Kim, Youngeun Lee.

**Project administration:** Jung-Ae Kim.

**Validation:** JiEun Kim, Jung-Ae Kim.

**Writing – original draft:** JiEun Kim, Min Young Kim, Jung-Ae Kim.

**Writing – review & editing:** JiEun Kim, Min Young Kim, Jung-Ae Kim, Youngeun Lee.

## Supplementary Material


