# Supplementary material for: Factors affecting preventive behaviors of Alzheimer’s disease in family members of patients with Alzheimer’s disease
Source: Medicine (Baltimore). 2022 Oct 21;101(42):e31136. doi: 10.1097/MD.0000000000031136 (PMC9592491; doi:10.1097/MD.0000000000031136)
Supplement: Supplementary file 1 [file medi-101-e31136-s001.pdf]

Supplemental Digital Content 1. Score of Alzheimer's disease (AD) preventive lifestyle according to the intention and prior experience

AD-preventive lifestyle	Total	Intention to take AD-preventive medicines			Experience of taking Gingko biloba or similar medicines		
		Yes	No	p-value	Yes	No	p-value
**n (% to total N)**	**147 (100.0%)**	**139 (94.6%)**	**8 (5.4%)**	**-**	**68 (46.3%)**	**79 (53.7%)**	**-**
**Total score**	**33.2 (4.9)**	**33.3 (4.8)**	**30.5 (4.6)**	**0.111†**	**33.8 (4.7)**	**32.6 (4.9)**	**0.118†**
Meal regularity	3.6 (1.0)	3.6 (1.0)	3.1 (1.1)	0.186†	3.6 (1.0)	3.5 (0.9)	0.784†
Balanced nutrition	3.7 (0.9)	3.7 (0.9)	3.5 (0.8)	0.512†	3.9 (0.9)	3.6 (0.9)	0.053†
Stop drinking	3.8 (1.3)	3.8 (1.3)	3.9 (1.4)	0.857†	3.9 (1.2)	3.8 (1.3)	0.614†
Smoking cessation	4.4 (1.1)	4.5 (1.1)	4.3 (1.2)	0.598†	4.4 (1.1)	4.5 (1.0)	0.632†
Body weight management	3.5 (1.0)	3.5 (1.0)	3.3 (0.7)	0.527†	3.6 (0.9)	3.4 (1.0)	0.201†
Regular exercise (>10 min/day)	3.4 (1.1)	3.4 (1.1)	2.8 (1.2)	0.096†	3.5(1.2)	3.3 (1.1)	0.201†
Regular intellectual activities (reading, watching movie, etc.)	3.2 (1.1)	3.2 (1.0)	2.9 (1.0)	0.338†	3.3 (1.0)	3.1 (1.1)	0.252†
Regular management of chronic disease	3.6 (0.9)	3.7 (0.9)	2.8 (0.9)	0.005†	3.8 (0.7)	3.5 (1.0)	0.042†
Frequent contacts with family member or friends	4.0 (0.7)	4.0 (0.7)	4.1 (0.4)	0.236†	3.9 (0.7)	4.0 (0.7)	0.780†

Values are presented as mean (SD).
†T-test
AD, Alzheimer's disease; SD, Standard deviation.
